# Factors associated with life-space mobility in older adults with chronic respiratory disease: IMIAS study

**DOI:** 10.3389/fresc.2026.1775074

**Published:** 2026-06-10

**Authors:** Ala’ S. Aburub, Ricardo Oliveira Guerra, Nailton José Neto, Aseel Aburub, Mohammad Z. Darabseh, Motaz Alawna, Marta Hock, Viktória Prémusz

**Affiliations:** 1 Department of Rehabilitation Sciences, Faculty of Applied Medical Sciences, Jordan University of Science and Technology, Irbid, Jordan; 2 Physiotherapy Department, Federal University of Rio Grande do Norte, Natal, Brazil; 3 Department of Physiotherapy, Federal University of Rio Grande do Norte, Natal, Brazil; 4 Department of Physiotherapy, Faculty of Allied Medical Sciences, Applied Science Private University, Amman, Jordan; 5 Department of Physiotherapy, School of Rehabilitation Sciences, The University of Jordan, Amman, Jordan; 6 Department of Health Sciences, Faculty of Graduate Studies, Arab American University, Jenin, Palestine; 7 Institute of Physiotherapy and Sports Science, Faculty of Health Sciences, University of Pécs, Pécs, Hungary; 8 Physical Activity Research Group, János Szentágothai Research Center, University of Pécs, Pécs, Hungary; 9 National Laboratory on Human Reproduction, University of Pécs, Pécs, Hungary

**Keywords:** chronic respiratory diseases (CRD), life space mobility (LSM), older adults, physiotherapy, restricted and unrestricted life space mobility

## Abstract

**Introduction:**

Life space mobility (LSM) reflects overall function, and a reduction in LSM can limit activity, participation, and quality of life in older adults with chronic respiratory diseases (CRD). Existing research has not fully addressed factors associated with LSM. This study examined demographic, clinical, financial, social, and environmental factors associated with LSM in older adults living with CRD and compared characteristics of those with restricted versus unrestricted LSM.

**Method:**

Participants completed the Life-Space Assessment (LSA) as the primary outcome, along with questionnaires collecting demographic, financial, social, environmental, and clinical data. LSA scores ranged from 0–120, with a cutoff of ≤ 60 used to identify restricted LSM. LSM groups (restricted vs unrestricted) were contrasted using chi-square tests and t-tests. Factors associated with LSM were analyzed using both simple linear and multiple linear.

**Results:**

Among 223 CRD participants (103 restricted LSM, 120 unrestricted), groups differed in demographics (study site, sex, and education), all financial variables (*p* < 0.05), and clinical variables, including depression, visual acuity, cognition, fear of falling, physical function, self-rated health, and grip strength (*p* < 0.05). Adjusted regression showed sex, self-rated health, vision, and physical performance significantly predicting LSM (*p* < 0.05).

**Conclusion:**

LSM is a multifactorial factor associated with socio-demographic and functional factors in older adults with CRD.

## Introduction

Chronic respiratory disease (CRD) is defined as inflammation of the lung airways and includes several conditions, such as chronic obstructive pulmonary disease (COPD), asthma, interstitial lung disease, pneumoconiosis, and others (1). As the population is aging, the number of individuals diagnosed with CRD has increased since 1990 (1, 2). A study conducted in 2019 reported that worldwide, around 454 million people were diagnosed with CRD, and almost 213 million of them were diagnosed with COPD (1). Furthermore, CRD has been the third leading cause of death worldwide since 2019 (1). A forecast analysis predicts that the number of affected individuals will continue to increase by 2035 (3), highlighting the growing burden on healthcare systems.

Complications of CRD extend beyond damaging the lungs to impact individuals at multiple system levels, as well as their psychological and physical health. System-level complications include pulmonary hypertension (4), cardiovascular disease, diabetes, and lung cancer (5). Anxiety and depression are examples of common psychological complications, with prevalence among people with COPD ranging between 31 and 46% (6). Physical complications include diminished physical function (hindering daily activities) and reduced life-space mobility (LSM), which subsequently declines overall quality of life (7–9).

Life-space mobility (LSM) reflects overall function by measuring a person's ability to move indoors (e.g., home) and outdoors (e.g., neighborhood, city), as well as reporting the frequency and independence of these movements, including the use of assistive devices (10). Research among older adults has shown that deterioration in LSM links to decreased physical activity, increased depressive symptoms, restricted social participation, and lower quality of life (QOL) (11–13).

In people with CRD, relatively few studies have investigated LSM in older adults (7–11). One study has explored the association of LSM and the following variables: dyspnea, peripheral muscle strength, and physical inactivity (7). Another study has investigated the links between LSM and clinical outcomes, including depression, anxiety, hospital and emergency visits, walking, dyspnea, and QOL in older adults with COPD (10). Previous studies have also examined the relationship between LSM and factors such as dyspnea severity, activities of daily living (ADL) (8), grip strength, walking, respiratory function, and knee extension strength (9), comorbidities, exercise capacity, and QOL (11).

Although the prior studies provide valuable insights (7–11). Several demographics, clinical, financial, social, and environmental factors remain unexplored in older adults with CRD. Therefore, to address this gap, the present study aimed to investigate the association between demographic, clinical, financial, social, and environmental factors and LSM in older adults living with CRD and to compare characteristics of those with restricted versus unrestricted LSM.

To our knowledge, no previous study has comprehensively examined demographic, financial, social, environmental, and clinical correlates of LSM simultaneously in older adults with CRD using multinational IMIAS data. Identifying factors associated with LSM may provide insight into mobility limitations, inform the development of targeted interventions, help prevent functional decline, and support healthcare planning and policy.

## Method

### Participants and study design

This was a cross-sectional secondary analysis of data from the 2012 IMIAS cohort. Participants aged 65–74 years diagnosed with chronic respiratory disease (CRD) were drawn from the 2012 International Mobility in Aging Study (IMIAS) across four countries (12). Inclusion criteria included participants aged between 65 and 74 years who reported a physician-diagnosed chronic respiratory disease and completed the Life-Space Assessment (LSA). CRD was defined based on self-report of a physician-diagnosed condition. Participants were classified as having CRD if they reported any of the following chronic lung conditions: chronic obstructive pulmonary disease (COPD), asthma, or chronic bronchitis. Exclusion criteria included participants with ≥ 4 errors on the Leganés Cognitive Test (LCT), inability to comply with study procedures, or incomplete data related to the primary study variables (13).

### Outcome variables and determinants

Participants completed the Life-Space Assessment (LSA) as the primary outcome, along with questionnaires collecting demographic, financial, social, environmental, and clinical data.

LSA evaluates three aspects of individuals’ mobility capacity, frequency, and independence across nine areas, scoring 0–120, with higher scores reflecting greater mobility, and has been validated in older adults (14, 15). Various cutoff points define restricted LSM; however, the cutoff point of LSM in people with various CRD is not standardized, but previous studies used ≤ 60 to define restricted LSM in people with COPD (10, 11). A cutoff similar to previous studies was adopted in this analysis.

The demographic variables examined included study location, age, sex, and education. The financial characteristics examined were work types, received income, and adequacy of income. The social and environmental variables included living alone and social support received by participants’ close circle, including family, children, partner, and friends (0–20, higher score = greater support).

The clinical variables examined participants’ depressive symptoms, visual acuity, cognitive status, fear of falling (FOF), physical performance, self-rated health, number of comorbidities, body mass index, and grip strength.

Center for Epidemiological Studies Depression (CESD) evaluated depression with scores ranging from 0 to 60 (higher = more severe symptoms) (12, 13, 16); the ETDRS tumbling-E chart for visual acuity, 0–70 (higher = better vision) (17); LCT for cognitive status, 0–32 (higher = better cognitive status); Falls Efficacy Scale International (FES-I) for FOF, 16–64 (higher = more fears) (18); and the Short Physical Performance Battery (SPPB) for physical performance, 0–12 (higher = greater physical function) (19). These measures have demonstrated validity and reliability across multiple populations (13, 16, 18–23). BMI was classified into four categories from Underweight (< 18.5) to Obesity (> = 30) (24). Grip strength was recorded as the best value among the three trials using an A Jamar handheld dynamometer (25). Comorbidities were classified as 1, 2, or ≥ 3 conditions.

### Statistical analysis

All study variables were summarized descriptively, using appropriate descriptive statistics for continuous and categorical data. Life space mobility groups (restricted vs unrestricted) were contrasted using chi-square tests (categorical data) and t-tests (continuous data).

Simple linear regression analyses were first conducted to examine the association between each independent variable and Life-Space Mobility (LSM). Variables that were statistically significant at *p* < 0.05 in the simple regression analyses were then entered into a multiple linear regression model. This approach was used to identify candidate predictors for multivariable modeling while limiting model overfitting and is commonly applied in exploratory observational studies. Dummy variables were created for all categorical predictors, with the first category serving as the reference group in all regression models. Reference categories used in the regression models were as follows: study site (Kingston), sex (male), education (primary level), work type (manual), income status (receiving income: yes), income sufficiency (very well), self-rated health (good/very good), and number of comorbidities (one).

A final multiple linear regression model was then constructed by additionally adjusting for age, sex, and study site to control for potential confounding.

Regression results are presented as beta coefficients with 95% confidence intervals (95% CI). The overall model fit was assessed using the coefficient of determination (*R*^2^). All analyses were performed using SPSS version 26.

## Results

As presented in Table 1, 223 participants with CRD were analyzed. Of these, 103 demonstrated restricted LSM, while 120 showed no restriction according to LSA scores, along with their characteristics and results from chi-square and t-test. Mean age did not differ between LSM groups (restricted 69.3 ± 2.8; unrestricted 68.9 ± 2.95; t-1.02, *p* = 0.31). Demographic variable-Study sites, sex, and education- differed significantly between LSM groups as reflected in chi-square statistics of 27.37, 12.32, and 11.61, respectively; *p* < 0.05. LSM groups differed significantly in all financial variables (*p* < 0.05). Moreover, no social or environmental factors demonstrated statistically significant variation across LSM groups. Participants in restricted vs. unrestricted LSM groups differed significantly (*p* < 0.05) in depression, visual acuity, cognition, fear of falling, physical function, self-rated health, and grip strength as reflected in chi-square and t-test statistics.

**Table 1:** Participants’ characteristics with CRD were analyzed using the chi-square or t-test.

	Older adults with chronic lung disease			
Demographic variables	Restricted LSM < = 60	non-restricted LSM > 60	Chi-square (*Χ*²)/t-test	*P*-value
Study site	*N* (%)			
Kingston (Canada)	10 (9.7)	40 (33.3)	*Χ*² (27.37)	< 0.001*
St. Hyacinthe (Canada)	14 (13.6)	24 (20)		
Tirana (Albania)	27 (26.2)	20 (16.7)		
Manizales (Colombia)	28 (27.2)	27 (22.5)		
Natal (Brazil)	24 (23.3)	9 (7.5)		
Age (years)	103 (69.3 ± 2.8)	120 (68.9 ± 2.95)	1.03	0.31
Age category (years)				
64–69	54 (52.4)	70 (58.3)	*Χ*² (0.78)	0.37
70–75	49 (47.6)	50 (41.7)		
Gender				
Male	27 (26.2)	59 (49.2)	*Χ*² (12.32)	< 0.001*
Female	76 (73.8)	61 (50.8)		
Education level				
Primary	67 (65)	52 (43.7)	*Χ*² (11.61)	0.003*
Secondary	11 (10.7)	13 (10.9)		
post-secondary	25 (24.3)	54 (45.4)		
Marital status				
Single (never married)	8 (7.8)	6 (5)	*Χ*² (5.81)	0.12
Married - common law	49 (47.6)	73 (60.8)		
Widow -widower	29 (28.2)	20 (16.7)		
Separated - divorced	17 (16.5)	21 (17.5)		
Financial variables				
Type of work				
Manual	81 (80.2)	63 (52.5)	*Χ*² (18.53)	< 0.001*
Non-Manual	20 (19.8)	57 (47.5)		
Receiving income				
Yes	91 (88.3)	116 (96.7)	*Χ*² (5.75)	0.016*
No	12 (11.7)	4 (3.3)		
Income sufficiency				
Very well	5 (4.9)	38 (31.9)	*Χ*² (27.13)	< 0.001*
suitable	32 (31.4)	34 (28.6)		
Not very well	65 (63.7)	47 (39.5)		
Social and Environmental variables				
Living alone				
Yes	26 (25.24)	27 (22.5)	*Χ*² (0.23)	0.63
No	77 (74.6)	93 (77.5)		
Social support (friends), score (0–20)	75 (16.01 ± 3.286)	97 (16.09 ± 3.56)	−0.15	0.881
Social support (children), score (0–20)	92 (16.5 ± 4.3)	113 (17.1 ± 3.6)	−1.143	0.254
Social support (family), score (0–20)	102 (14.7 ± 4.5)	120 (14.9 ± 4.6)	−0.36	0.72
Social support (partner), score (0–20)	50 (17.7 ± 3.0)	76 (3.0 ± 2.9)	−1.027	0.307
Clinical Variables				
Depressive symptoms (CES-D), score (0–60)	103 (18.5 ± 13.3)	118 (9.4 ± 9.4)	5.883	< 0.001*
Visual acuity (ETDRS Tumbling E), score (0–70)	101 (37.8 ± 12.4)	117 (44.9 ± 10.9)	−4.518	< 0.001*
Cognitive status (Leganés Cognitive Test), score (0–32)	103 (27.8 ± 2.8)	120 (28.6 ± 2.7)	−2.152	0.032
Fear of falling (FES-I), score (16–64)	103 (32.5 ± 13.6)	120 (23.5 ± 8.7)	5.946	< 0.001*
Physical performance (SPPB), score (0–12)	103 (7.6 ± 2.7)	120 (9.9 ± 1.71)	−7.886	< 0.001*
Have you fallen in the last 12 months?	103 (1.58 ± 0.51)	120 (1.68 ± 0.47)	−1.441	0.151
Number of falls				
Never	58 (58)	80 (67.8)	*Χ*² (4.58)	0.21
One	18 (18)	16 (13.6)		
Two	11 (11)	15 (12.7)		
Three or more	13 (13)	7 (5.9)		
Self-health rated				
Good/very good	20 (19.4)	66 (55)	*Χ*² (29.6)	< 0.001*
Fair/poor/very poor	83 (80.6)	54 (45)		
Number of comorbidities				
one	5 (4.9)	16 (13.3)	*Χ*² (4.58)	0.2
Two	23 (22.3)	33 (27.5)		
three or more	75 (72.8)	71 (59.2)		
Body Mass Index				
Underweight (< 18.5)	2 (1.9)	7 (5.8)	*Χ*² (3.98)	0.26
Normal (18.5–24.9)	30 (29.1)	29 (24.2)		
Overweight (25–29.9)	33 (32)	47 (39.2)		
Obesity (> = 30)	38 (36.9)	37 (30.8)		
Max grip strength in KG	99 (21.6 ± 9.0)	119 (29.1 ± 10.1)	−5.72	< 0.001*

Values are presented as mean ± standard deviation (SD) or n (%). Chi-square tests were used for categorical variables, and independent t-tests were used for continuous variables. Statistical significance was set at *p* < 0.05.

CES-D, Center for Epidemiological Studies depression scale; SPPB, short physical performance battery; LCT, Leganés cognitive test; FES-I, falls efficacy scale–international; kg, kilograms; SD, standard deviation.

*p < 0.05.

Table 2 summarizes the simple linear regression analysis of factors linked to LSM. Study site, sex, and education emerged as significant demographic predictors of LSM (*p* < 0.05). LSM was significantly predicted by all financial variables (*p* < 0.05). LSM was significantly (*p* < 0.05) predicted by the following clinical variables: depression, visual acuity, cognition, fear of falling, physical function, self-rated health, grip strength, and number of comorbidities.

**Table 2:** Simple linear regression results for life-space mobility among older adults with chronic respiratory diseases (*N* = 223).

Study variables	Unstandardized B	Standard error	T-test	Pr > |t|
Demographic variables				
Study site	−4.73	1.04	−4.56	< 0.001*
Age	−0.77	0.51	−1.5	0.135
Sex	−14.72	2.91	−5.1	< 0.001*
Marital status	−1.43	1.77	−0.81	0.418
Education	2.14	0.46	4.64	< 0.001*
Financial variables				
Type of work	14.6	3.0	4.87	< 0.001*
Receiving income	−13.58	5.72	−2.37	0.018*
Income sufficiency	−10.28	1.81	−5.67	< 0.001*
Social and environmental variables				
Living alone	0.36	3.51	0.10	0.918
Social support (friends), score (0–20)	0.33	0.48	0.69	0.492
Social support (children), score (0–20)	0.56	0.40	1.39	0.167
Social support (family), score (0–20)	0.06	0.33	0.19	0.845
Social support (partner), score (0–20)	1.25	0.67	1.88	0.062
Clinical variables				
Depressive symptoms (CES-D), score (0–60)	−0.74	0.11	−6.51	< 0.001*
Visual acuity (ETDRS Tumbling E), score (0–70)	0.69	0.12	5.91	< 0.001*
Cognitive status (Leganés Cognitive Test), score (0–32)	1.80	0.52	3.44	0.001
Fear of falling (FES-I), score (16–64)	−0.86	0.11	−7.76	0.000*
Physical performance (SPPB), score (0–12)	5.17	0.49	10.61	< 0.001*
Have you fallen in the last 12 months?	5.21	3.04	1.71	0.088
Number of falls in the last year	−2.88	1.48	−1.94	0.053
Self-health rated	−20.18	2.76	−7.32	< 0.001*
Max grip strength in KG	0.99	0.13	7.65	< 0.001*
Body Mass Index	−0.97	1.71	−0.57	0.572
Number of co-morbidities	−4.65	2.25	−2.07	0.04*

CES-D, Center for Epidemiological Studies depression scale; ETDRS, early treatment diabetic retinopathy study; FES-I, falls efficacy scale–international; SPPB, short physical performance battery; kg, kilograms.

* Statistical significance was set at *p* < 0.05.

Non-adjusted multiple linear regression analyses are summarized in Table 3. Sex was significantly associated with LSM (*B* = −7.73, *t* = −2.29, *p* = 0.023, 95% CI [−14.39, −1.06]). Similarly, vision (*B* = −15.96, *t* = −3.15, *p* = 0.002, 95% CI [−25.96, −5.97]), physical performance (*B* = 2.98, t = 4.90, *p* = < 0.001, 95% CI [1.78, 4.18]), and self-rated health (*B* = −8.85, *t* = −3.26, *p* = 0.001, 95% CI [−14.21, −3.49]) were significantly associated with LSM. In the multivariable linear regression analysis, the model accounted for 53% of the variability in the outcome variable (*R*^2^ = 0.53), with a slightly lower adjusted *R*^2^ of 0.50, suggesting a good overall model fit with limited inflation from included predictors.

**Table 3:** Non-adjusted multiple linear regression results for life-space mobility among older adults with chronic respiratory diseases (*N* = 223).

Study variables	Unstandardized B	Standard error	T-test	Pr > |t|	95% CI lower	95% CI upper
Study site	−1.99	1.18	−1.70	0.092	−4.31	0.33
Sex	−7.73	3.38	−2.29	0.023*	−14.39	−1.06
Education	−1.43	1.77	−0.81	0.420	−4.93	2.07
Work Types	6.53	3.44	1.90	0.059	−0.25	13.31
Do you receive an income?	−1.59	4.55	−0.35	0.727	−10.57	7.38
Income Sufficient	0.81	2.00	0.41	0.686	−3.14	4.76
Depressive symptoms (CES-D), score (0–60)	−0.19	0.11	−1.69	0.093	−0.41	0.03
Visual acuity (ETDRS Tumbling E), score (0–70)	−15.96	5.07	−3.15	0.002*	−25.96	−5.97
Cognitive status (Leganés Cognitive Test), score (0–32)	−0.61	0.46	−1.32	0.189	−1.52	0.30
Fear of falling (FES-I), score (16–64)	−0.17	0.12	−1.40	0.163	−0.41	0.07
Physical performance (SPPB), score (0–12)	2.98	0.61	4.90	< 0.001*	1.78	4.18
Self-rated health	−8.85	2.72	−3.26	0.001*	−14.21	−3.49
Max grip strength in KG	0.08	0.18	0.43	0.671	−0.28	0.43
Number of co-morbidities	2.25	1.85	1.21	0.227	−1.41	5.91

CES-D, Center for Epidemiological Studies depression scale; ETDRS, Early Treatment Diabetic Retinopathy Study; FES-I, Falls Efficacy Scale–International; SPPB, Short Physical Performance Battery; kg, kilograms.

* Statistical significance was set at *p* < 0.05; 95% Confidence Interval; *R*² = 0.53, adjusted *R*² = 0.50.

Adjusted multiple linear regression analysis, controlling for age, gender, and study site, confirmed that sex, vision, physical performance, and self-rated health were significant predictors of LSM (*p* < 0.05) (Table 4). The multivariable linear regression model explained 53% of the variance in the outcome variable (*R*^2^ = 0.53), with an adjusted *R*^2^ of 0.50.

**Table 4:** Adjusted multiple linear regression results for life-space mobility among older adults with chronic respiratory diseases (*N* = 223).

Study variables	Unstandardized B	Standard error	T-test	Pr > |t|	95% CI lower	95% CI upper
Age	−0.62	0.39	−1.59	0.113	−1.38	0.15
Study site	−2.09	1.17	−1.78	0.076	−4.40	0.22
Sex	−7.66	3.37	−2.27	0.024*	−14.30	−1.02
Education	−1.62	1.77	−0.92	0.360	−5.11	1.87
Work Types	6.47	3.42	1.89	0.060	−0.29	13.22
Do you receive an income?	−1.37	4.53	−0.30	0.763	−10.31	7.57
Income Sufficient	0.51	2.00	0.26	0.797	−3.43	4.46
Depressive symptoms (CES-D), score (0–60)	−0.21	0.11	−1.86	0.064	−0.43	0.01
Visual acuity (ETDRS Tumbling E), score (0–70)	−15.12	5.07	−2.98	0.003*	−25.12	−5.11
Cognitive status (Leganés Cognitive Test), score (0–32)	−0.59	0.46	−1.28	0.201	−1.50	0.32
Fear of falling (FES-I), score (16–64)	−0.16	0.12	−1.27	0.204	−0.40	0.09
Physical performance (SPPB), score (0–12)	2.95	0.61	4.87	< 0.001*	1.75	4.14
Self-rated health	−9.02	2.71	−3.33	0.001*	−14.36	−3.68
Max grip strength in KG	0.07	0.18	0.40	0.689	−0.28	0.43
Number of co-morbidities	2.40	1.85	1.30	0.196	−1.25	6.05

CES-D, Center for Epidemiological Studies depression scale; ETDRS, Early Treatment Diabetic Retinopathy Study; FES-I, Falls Efficacy Scale–International; SPPB, Short Physical Performance Battery; kg, kilograms.

* Statistical significance was set at *p* < 0.05; 95% CI: Confidence Interval; *R*² = 0.53, adjusted *R*² = 0.50.

**Figure 1:**
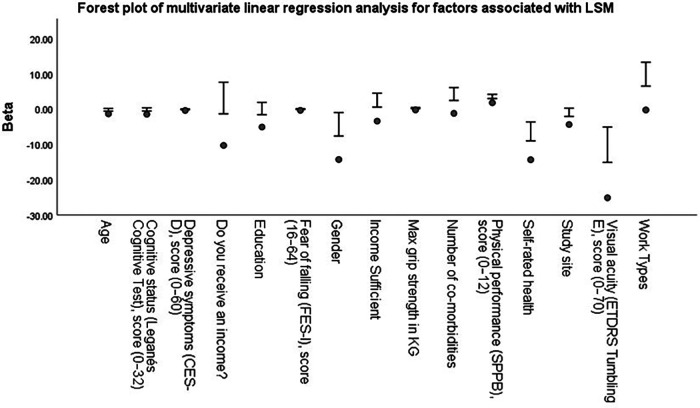
Forest plot of the final adjusted multiple linear regression model examining factors associated with life-space mobility among older adults.

A forest plot of the final adjusted multiple linear regression model examining factors associated with life-space mobility among older adults is shown in Figure 1. Female gender, poorer visual acuity, poorer physical performance, and poorer self-rated health were significantly associated with life-space mobility.

Female participants had significantly lower life-space mobility scores compared with males (*B* = −7.66, 95% CI: −14.30–−1.02). Similarly, participants with poor self-rated health had lower life-space mobility scores compared with those reporting good self-rated health (*B* = −9.02, 95% CI: −14.36–−3.68). Visual acuity was also significantly associated with life-space mobility (*B* = −15.12, 95% CI: −25.12–−5.11), indicating that poorer visual acuity was associated with reduced mobility. In contrast, better physical performance, measured using the SPPB, was positively associated with life-space mobility (*B* = 2.95, 95% CI: 1.75–4.14).

Other variables, including age, study site, education, work type, income status, depressive symptoms, cognitive status, fear of falling, grip strength, and number of comorbidities, were not significantly associated with life-space mobility in the final adjusted model, as their confidence intervals crossed zero.

## Discussion

The study aimed to explore how demographic, clinical, financial and social/environmental factors influence LSM in older adults living with CRD and to compare characteristics of those with restricted versus unrestricted LSM.

In this study, sex was the only demographic variable that significantly predicted LSM in older adults with CRD. Among the clinical factors, vision, physical performance, and self-rated health were identified as significant predictors. These findings emphasize the multifactorial nature of mobility in this population, in which both socio-demographic characteristics and functional health status influence older adults’ mobility in their living environment.

Our result that sex predicts LSM is consistent with prior research in both healthy older adults and in individuals with CRD (8, 26). Particularly, a study among older adults with COPD confirmed that women tend to have lower LSA scores, indicating more restricted LSM compared to men, which supports sex differences in mobility (8). Several reasons may explain these sex differences. Previous studies in older populations and in people with COPD have shown that women generally exhibit lower muscle mass, greater fear of falling, more severe COPD symptoms such as severe dyspnea, poor quality of life, a higher prevalence of frailty, and greater functional limitations- all of which are associated with greater life space restriction (8, 27–32).

Our study revealed that vision is associated with LSM. To date, no studies have directly established the link between vision and LSM in older adults with CRD. However, studies in older adults have reported that vision disorders are associated with reduced physical function, increased likelihood of falling, greater fear of falling, increased physical and mental comorbidities, increased frailty, and decreased overall functioning (33–40). Two studies in healthy older adults showed that vision impairments contribute to limited LSM and social participation (38, 39). In people with CRD, visual impairments may further compound existing challenges such as dyspnea and muscle deconditioning, making it more difficult to navigate their environment and leading to greater restrictions in LSM.

Physical performance was identified as a significant predictor of LSM in our study. A moderate correlation was found between greater restriction in LSM and both reduced peripheral muscle strength and lower physical activity in older adults with COPD (7). Positive associations have also been found in a recent study in older adults with COPD on long oxygen therapy, where LSM was associated with walking capacity measured by the 6-minute walk test (11). A study comparing individuals with COPD to a control group reported that gait abnormalities limit LSM, reducing both movement and stability (41, 42). Similarly, findings from the International Mobility in Aging Study (IMIAS) demonstrated that physiological burden and reduced physical performance are closely associated with mobility limitation and functional decline among older adults, further supporting the multidimensional nature of mobility restriction in aging populations (42).

Self-health rating (SRH) was another predictor of LSM in our study. SRH reflects individuals’ perception of their overall health in different aspects, including physical, psychological, and functional well-being (43–45). Therefore, poor SRH has been associated with cognitive decline, higher mobility disability, reduced confidence in performing exercise or daily activities, and limited social participation in healthy older adults (46–48). Similar findings have been reported in older adults with COPD, where poor SHR contributes to greater mobility limitation, poor quality of life, more severe depression and anxiety, poor self-management, increased symptom burden, higher rates of exacerbations and hospitalization, and limited social participation (49–57). These complications observed in both healthy populations and older adults with COPD help to explain why SRH predicts LSM in older adults with CRD.

The other aim was to compare characteristics of older adults with CRD who have restricted versus unrestricted LSM. Sex and education differed significantly between both LSM groups. Differences in LSM based on sex have been previously reported in older adults with COPD (8, 27, 28, 30, 31). Differences in education between LSM groups may reflect a vicious cycle, where lower education is linked to poor disease management, increased exacerbations or functional decline, and consequently restricted LSM. Furthermore, our study showed that LSM groups differed significantly in all financial variables (work types, receiving income, and income sufficiency). No study has examined directly the differences in financial status between older adults with CRD who have restricted versus unrestricted LSM. However, evidence from the general older population suggests that socioeconomic status predicts LSM (58, 59), and lower occupational status and insufficient income are associated with greater functional limitation (60, 61). In people with CRD, financial burden may both restrict mobility and result from limited life-space.

Our study also revealed that, on average, participants with restricted LSM had worse depressive symptoms, visual acuity, cognitive status, greater fear of falling, reduced physical performance, poor SRH, and weaker grip strength compared to those with unrestricted LSM. These findings are aligned with prior research in the general population and older adults with COPD (7, 18, 40, 44, 49, 55, 56, 62).

In summary, sex, vision, physical performance, and self-rated health each play a significant role in LSM among older adults with CRD. Sex reflects biological and psychosocial differences, vision influences safety and navigation, physical performance determines functional capacity, and SRH captures perceived health and confidence. The observed differences between restricted and unrestricted LSM groups highlight that mobility limitations are multifactorial, involving physical, cognitive, psychological, and sensory factors. Therefore, effective interventions should adopt a multidimensional approach, including physical rehabilitation, cognitive and vision support, fall prevention strategies, and mental health interventions.

The findings of this study have important clinical implications for the assessment and management of older adults with chronic respiratory disease. Identifying factors associated with restricted life-space mobility may help healthcare professionals recognize individuals at increased risk of functional decline, reduced community participation, and poorer quality of life. Incorporating life-space mobility assessment into routine clinical evaluation may support early identification of mobility limitations and guide individualized rehabilitation interventions targeting physical performance, vision-related difficulties, fall prevention, and self-management strategies. Furthermore, the findings support the importance of adopting multidimensional rehabilitation approaches that address not only physical impairments but also sensory, psychosocial, and health-perception factors that may contribute to mobility restriction.

From a theoretical perspective, this study contributes to the growing understanding that life-space mobility is a complex and multidimensional construct influenced by the interaction of biological, functional, psychological, and environmental factors. The present findings further support conceptual models of mobility in aging populations by demonstrating that mobility restriction in older adults with chronic respiratory disease cannot be explained solely by respiratory impairment, but rather by the combined influence of multiple health and functional domains.

As this study employed a cross-sectional design, causal inferences cannot be made, and findings should be interpreted as associations only. The inclusion of participants with different chronic respiratory diseases may limit the specificity of conclusions for individual conditions. Furthermore, the findings are generalizable only to community-dwelling adults aged 65–74 years. Additional limitations include the use of secondary data, which may not have captured all relevant variables, and the presence of potential residual confounding due to unmeasured factors. CRD diagnosis and some study variables were based on self-report, which may introduce recall and misclassification bias. In particular, variables such as sleep disturbances and anxiety, which may influence life-space mobility, were not assessed. There is also the possibility of missing data affecting the analyses. In this study, LSM cutoff was derived from COPD studies and may not generalize equally across all CRD subtypes.

## Conclusion

Sex emerged as the only significant demographic predictor of LSM, whereas vision, physical performance, and self-rated health were the strongest clinical predictors. Together, these findings underscore that mobility in older adults with CRD is shaped by a complex interplay of biological, perceptual, and functional factors, emphasizing the need for integrated assessment and intervention strategies that go beyond demographic profiling alone.

## Data Availability

The data analyzed in this study is subject to the following licenses/restrictions: Data will be provided upon request. Requests to access these datasets should be directed to AA; asaburub@just.edu.jo.
